# Supplementary material for: Predicting visual function by interpreting a neuronal wiring diagram
Source: Nature. 2024 Oct 2;634(8032):113–23. doi: 10.1038/s41586-024-07953-5 (PMC11446822; doi:10.1038/s41586-024-07953-5)
Supplement: Supplementary file 6 — CRF and ERF predictions for individual TmY4 and TmY9 cells. Analogous to Supplementary Data 3, but for TmY target types. Shown are the top four monosynaptic pathways, the strongest pathway passing through each of the top ten intermediary types (ranking from Extended Data Fig. 7), and the trisynaptic pathway Tm1–TmY–Dm3–TmY (see the section entitled Prediction of spatial normalization). [file 41586_2024_7953_MOESM6_ESM.zip › DataS4/TmY9qΓèÑ/720575940640008765.pdf]

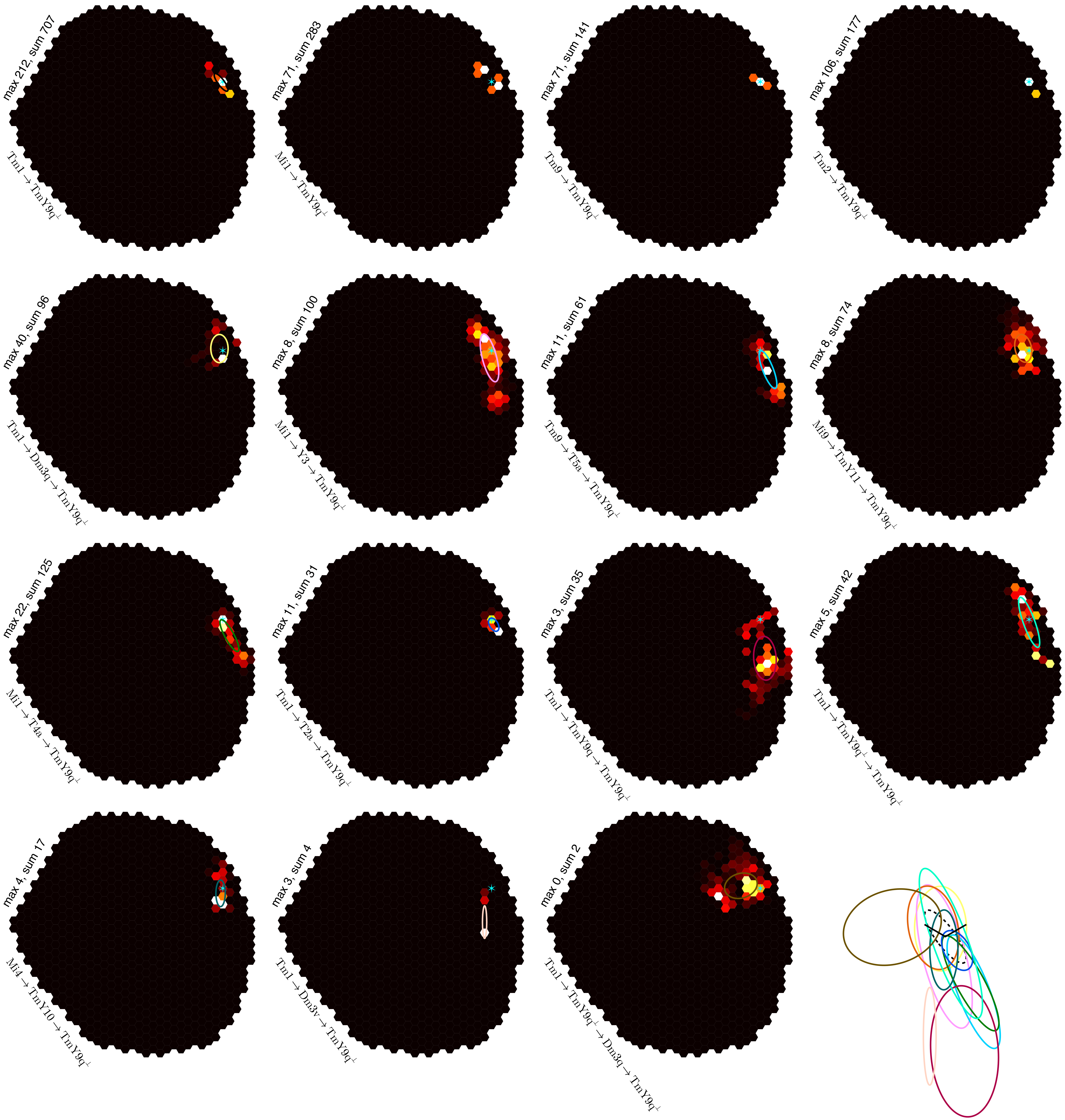

max 212, sum 707
Tm1 → TmY9q⊥
max 71, sum 283
Mi1 → TmY9q⊥
max 71, sum 141
Tm9 → TmY9q⊥
max 106, sum 177
Tm2 → TmY9q⊥
max 40, sum 96
Tm1 → Dm3q → TmY9q⊥
max 8, sum 100
Mi1 → Y3 → TmY9q⊥
max 11, sum 61
Tm9 → T5a → TmY9q⊥
max 8, sum 74
Mi9 → TmY11 → TmY9q⊥
max 22, sum 125
Mi1 → T4a → TmY9q⊥
max 11, sum 31
Tm1 → T2a → TmY9q⊥
max 3, sum 35
Tm1 → TmY9q → TmY9q⊥
max 5, sum 42
Tm1 → TmY9q⊥ → TmY9q⊥
max 4, sum 17
Mi4 → TmY10 → TmY9q⊥
max 3, sum 4
Tm1 → Dm3v → TmY9q⊥
max 0, sum 2
Tm1 → TmY9q⊥ → Dm3q → TmY9q⊥
